# Comparison of image quality between photon-counting detector CT and energy-integrating detector coronary CT angiography in heart transplant patients

**DOI:** 10.1007/s10554-025-03433-7

**Published:** 2025-06-02

**Authors:** Simran P. Sharma, Ricardo P.J. Budde, Jan Willem Groen, Marcel L. Dijkshoorn, Olivier C. Manintveld, Alexander Hirsch

**Affiliations:** 1 Department of Cardiology, Cardiovascular Institute, Thorax Center, Erasmus MC, Room Rg-419, Post Office Box: 2040, Rotterdam, 3000 CA The Netherlands; 2 Department of Radiology and Nuclear Medicine, Erasmus MC, Rotterdam, The Netherlands; 3 Erasmus MC Transplant Institute, Erasmus University Medical Center Rotterdam, Rotterdam, The Netherlands

**Keywords:** Heart transplantation, Computed tomography angiography, Coronary artery disease

## Abstract

This study aims to compare the image quality between photon-counting detector computed tomography (PCD-CT) and energy-integrating CT (EID-CT) for coronary artery visualization post-heart transplant (HTx) patients, using a paired comparison within one patient. Consecutive HTx patients who underwent both 3rd generation dual source EID-CT and ultra-high-resolution PCD-CT scans as part of routine clinical care, with a maximum interval of two years between scans were included. Image quality was assessed subjectively using a 5-point Likert scale and quantitatively with the signal-to-noise ratio (SNR) and contrast-to-noise ratio (CNR). Coronary segments were scored for presence, stenosis degree, presence of artifacts, and plaque composition. Thirty patients were included (mean age 50 ± 17 years; 21 (70%) men). The median interval between scans was 17[12–24] months. The median calcium score was 9[0–51] on the EID-CT scan and 25[0–92] on the PCD-CT. On the PCD-CT, 90% of coronary segments were rated very good or excellent compared to 71% on EID-CT (*p* < 0.001). Fewer segments were absent or non-evaluable due to size with PCD-CT compared to EID-CT (30% vs. 34%; *p* < 0.001). PCD-CT identified more mixed plaques (11% vs. 3%, *p* < 0.001). SNR and CNR were significantly higher on PCD-CT (22.1 ± 6.1 and 27.8 ± 6.8, *p* < 0.001) than on EID-CT (14.3 ± 3.4 and 16.9 ± 3.6, *p* < 0.001). The CT dose index volume was 6 [5-10] mGy on EID-CT and 19 [14-21] mGy on PCD-CT (*p* < 0.001). Ultra-high-resolution PCD-CT improves image quality compared to EID-CT in HTx patients. With superior SNR and CNR, and fewer non-evaluable segments, PCD-CT offers enhanced coronary artery visualization, though at the cost of an increased radiation dose.

**Supplementary Information:**

The online version contains supplementary material available at 10.1007/s10554-025-03433-7.

## Introduction

The International Society for Heart and Lung Transplantation recommends annual or biannual invasive coronary angiography to assess the development of cardiac allograft vasculopathy (CAV) [1]. CAV remains a significant cause of late morbidity and mortality in heart transplant (HTx) patients in whom early detection and monitoring of CAV are crucial for timely interventions that could improve patient outcomes [2]. However, non-invasive imaging modalities are gaining prominence due to the invasive nature and associated risks of invasive coronary angiography [3, 4].

Coronary computed tomography angiography (CCTA) has emerged as a valuable non-invasive alternative for evaluating coronary artery disease in HTx patients [3-5]. Photon-counting detector CT (PCD-CT) is the latest advancement in CT technology [6-8]. PCD-CT offers several advantages in CT technology over conventional energy-integrating detector CT (EID-CT), including higher spatial resolution, reduced blooming artifacts from high-attenuation objects such as stents and coronary calcifications, and decreased electronic noise [9-13]. These improvements may lead to better visualization of coronary arteries and plaques and a more accurate assessment of CAV in HTx patients [5].

Although various applications of PCD-CT have been studied, to our knowledge, no specific studies have evaluated its use in HTx patients [14-16]. Therefore, our study aimed to compare the image quality between PCD-CT and EID-CT for coronary artery visualization in HTx patients.

## Methods

### Study population

This retrospective study evaluated consecutive HTx patients who underwent a PCD-CT and a preceding EID-CT scan at the Erasmus MC, Rotterdam, the Netherlands, between September 2019 and January 2024. At Erasmus MC, patients routinely undergo annual CCTA assessment starting from the 5th year post-transplant for CAV detection. For all patients, the first scan was performed using a 3rd generation dual source EID-CT system (SOMATOM Force, Siemens Healthineers, Forchheim, Germany), followed by the PCD-CT scan using the NAEOTOM Alpha system (Siemens Healthineers, Forchheim, Germany) during subsequent annual follow-ups. Those scanned on any other EID-CT system or without informed consent were excluded from the study. The time interval between both scans was restricted to a maximum of two years to limit the influence of CAV progression. If multiple scans were available for a patient within the specified two-year timeframe, the two most recent scans with the shortest interval between them were selected for analysis to minimize the impact of CAV progression. The study was conducted in accordance with the Declaration of Helsinki and approved by the Medical Ethical Review Committee (MEC-2017-421) at Erasmus MC.

### Scan acquisition and reconstruction

Each patient initially underwent a non-contrast acquisition to assess coronary artery calcification scores. Following this, CCTA was conducted using prospective ECG triggering. For both scanners, automatic tube current modulation and tube voltage modulation were applied.

In patients with stable sinus rhythm and a heart rate ≤ 70 bpm, the ECG-pulsing window was configured to acquire images during the diastolic phase. For heart rates > 70 bpm, the pulsing window was adjusted to acquire the systolic phase. In patients with atrial fibrillation or arrhythmias, wide systolic and diastolic padding techniques were employed to ensure optimal imaging quality.

For EID-CT scans, the collimation was set to 96 × 0.6 mm with a 0.25-second rotation time. Image reconstruction for EID-CT used a slice thickness of 0.6 mm with 0.3 mm increments, employing two reconstruction kernels (Bv40, Bv49).

PCD-CT imaging was acquired using a collimation of 120 × 0.2 mm (ultra-high-resolution mode) with a 0.25-second rotation time. The image quality level was targeted at 65, with the tube current automatically adjusted to achieve this quality. Images were reconstructed with a 0.2 mm slice thickness and 0.15 mm increments, employing four different reconstruction kernels (Bv40, Bv44, Bv48, Bv56). The matrix size (512 × 512, 768 × 768, or 1024 × 1024 pixels) was chosen automatically to maintain pixel resolution.

Contrast media was administered using 70 mL of Iopromide/Ultravist 370 (Bayer Healthcare at a flow rate of 5.4 mL/sec for EID-CT scans and 85 ml of Iodixanol/Visipaque 320 (GE Healthcare at a flow rate of 5 ml/sec) for PCD-CT scans. A detailed overview of the scan protocols is presented in Supplementary Table 1.

### Image quality assessment

For the qualitative analysis, one radiologist (JG) with 2 years of experience in cardiovascular CT assessed each scan using the 18-segment model of the Society of Cardiovascular Computed Tomography [17]. The radiologist was blinded to the scanner type, and the scans were presented in a random order of either EID-CT or PCD-CT. Each coronary artery segment was initially classified as present, absent, or non-evaluable based on size. A segment was marked as “absent” if the image quality was deemed sufficient to determine that, if present, the segment would have been visible. Absent segments and segments that were non-evaluable due to size were excluded from further analysis.

Overall image quality was graded on a 5-point Likert scale, where 1 indicated poor quality, 2 adequate, 3 good, 4 very good, and 5 excellent. The radiologist also assessed each segment individually for the presence of motion artifacts, stack artifacts, and other artifacts (e.g., metal or stent-related), the presence of plaque (yes/no), plaque composition (calcified, non-calcified, mixed), and degree of diameter stenosis (0%, 1–24%, 25–49%, 50–69%, 70–89%, ≥ 90%, non-evaluable).

For the quantitative analysis, images reconstructed with the sharpest kernel available were selected. Regions of interest were placed in the ascending aorta and subcutaneous adipose tissue (SUB) to measure the mean and standard deviation (STD) of the CT values in Hounsfield Units. The region of interest was drawn as large as possible to improve reliability. From these values, signal-to-noise ratio (SNR) and contrast-to-noise ratio (CNR) were derived by the following equations:

1 $$S N R=\frac{M E A N_{\text {aorta }}}{S T D_{\text {aorta }}}$$

and

2 $$C N R=\frac{M E A N_{\text {aorta }}-M E A N_{\text {sub }}}{S T D_{\text {aorta }}}$$

### Statistical analysis

Categorical variables are expressed as frequencies and percentages. Continuous variables were expressed as mean ± STD or median with interquartile range, depending on the distribution. Normality was tested by using the Shapiro-Wilk test. The Wilcoxon Signed Rank test or paired samples t-test was employed to compare continuous variables. For the image quality, plaque composition and degree of stenosis comparisons, only coronary segments that were evaluable on both scans were included. McNemar’s test was used to compare categorical variables. Each plaque type (calcified, non-calcified or mixed) was assessed as a binary variable (present vs. not present) and compared between EID-CT and PCD-CT using the McNemar’s test. A Generalized Estimating Equations model was used to compare the image quality and degree of stenosis for the paired segments while accounting for the clustering of segments within individual patients. A two-tailed p-value of < 0.05 was considered statistically significant. All statistical analyses were performed using SPSS statistical software (IBM Corp. Released 2016. IBM SPSS Statistics for Windows, Version 28.0.1.0 Armonk; NY: IBM Corp.).

## Results

### Study population

A total of 30 HTx patients (mean age at the first scan 50 ± 17 years; 21 (70%) men; body mass index 26 [22–29] kg/m²) were included. The median interval between scans was 17 [12–24] months. The median time between HTx and the first scan on the EID-CT was 11 [9-15] years. The mean heart rate during EID-CT and PCD-CT scans was 81 ± 8 bpm and 78 ± 9 bpm (*p* = 0.313), respectively. Three patients had a history of percutaneous coronary intervention with stent placement before their first scan. Four patients had a pacemaker or implantable cardioverter-defibrillator; one received the device between the two scans, while the others had their devices implanted before the first scan. No other coronary interventions were performed between the two scans.

The median calcium score was 9 [0–51] on the EID-CT scan and 25 [0–92] on the PCD-CT (*p* < 0.001). The CT dose index volume was 6 [5-10] mGy on EID-CT and 19 [14-21] mGy on PCD-CT (*p* < 0.001). For EID-CT scans, the majority were acquired at 70 kV (*n* = 24), and a few at 80 kV (*n* = 4) or 90 kV (*n* = 2). PCD-CT scans were acquired at either 120 kV (*n* = 13) or 140 kV (*n* = 13), with only a few performed at 90 kV (*n* = 4). The patient characteristics and the radiation dose are presented in Table 1.

**Table 1:** Baseline and CT characteristics of included heart transplant patients

Patient characteristics	*N* = 30		
Male*	21 (70%)		
Age (years)*	50 ± 17		
Years after heart transplantation*	11 [9–15]		
Body mass index (kg/m^2^)*	26 [22–29]		
History of PCI with stent placement*	3 (10%)		
History of coronary artery bypass grafting*	0		
	**EID-CT** (***N = 30)***	**PCD-CT** (***N = 30)***	**P value**
Presence of pacemaker/ ICD	3 (10%)	4 (13%)	1.00
Heart rate (beats per minute)	81 ± 8	78 ± 9	0.539
Median calcium score**	9 [0–51]	25 [0–92]	< 0.001
Radiation dose			
CTDIvol (mGy)	6 [5–10]	19 [14–21]	< 0.001
DLP (mGy·cm)	79 [64–114]	283 [154–323]	< 0.001

Data is presented as mean ± Standard Deviation (SD), median [25th – 75th percentile], or frequencies (percentage). Dose Length Product (DLP); Implantable cardiac defibrillator (ICD); Percutaneous Coronary Intervention (PCI); Volumetric computed tomography dose index (CTDIvol) *At the time of the first scan, no PCI or coronary artery bypass grafting between scans. **Calcium scores of 27 patients (3 patients with a history of PCI were excluded)

### Subjective image quality assessment

A total of 1080 coronary segments (30 patients x 18 segments x 2 scans) were potentially available for evaluation. On the EID-CT scans, 183 (34%) of the 540 potential segments (30 patients x 18 segments) were classified as absent or non-evaluable due to size. On the PCD-CT scans, 163 segments (30%) were classified as absent or were non-evaluable due to size (*p* < 0.001). Image quality was assessed for segments that were evaluable on both scans, resulting in a total of 672 analysed segments (336 on EID-CT and 336 on PCD-CT).

**Fig. 1:**
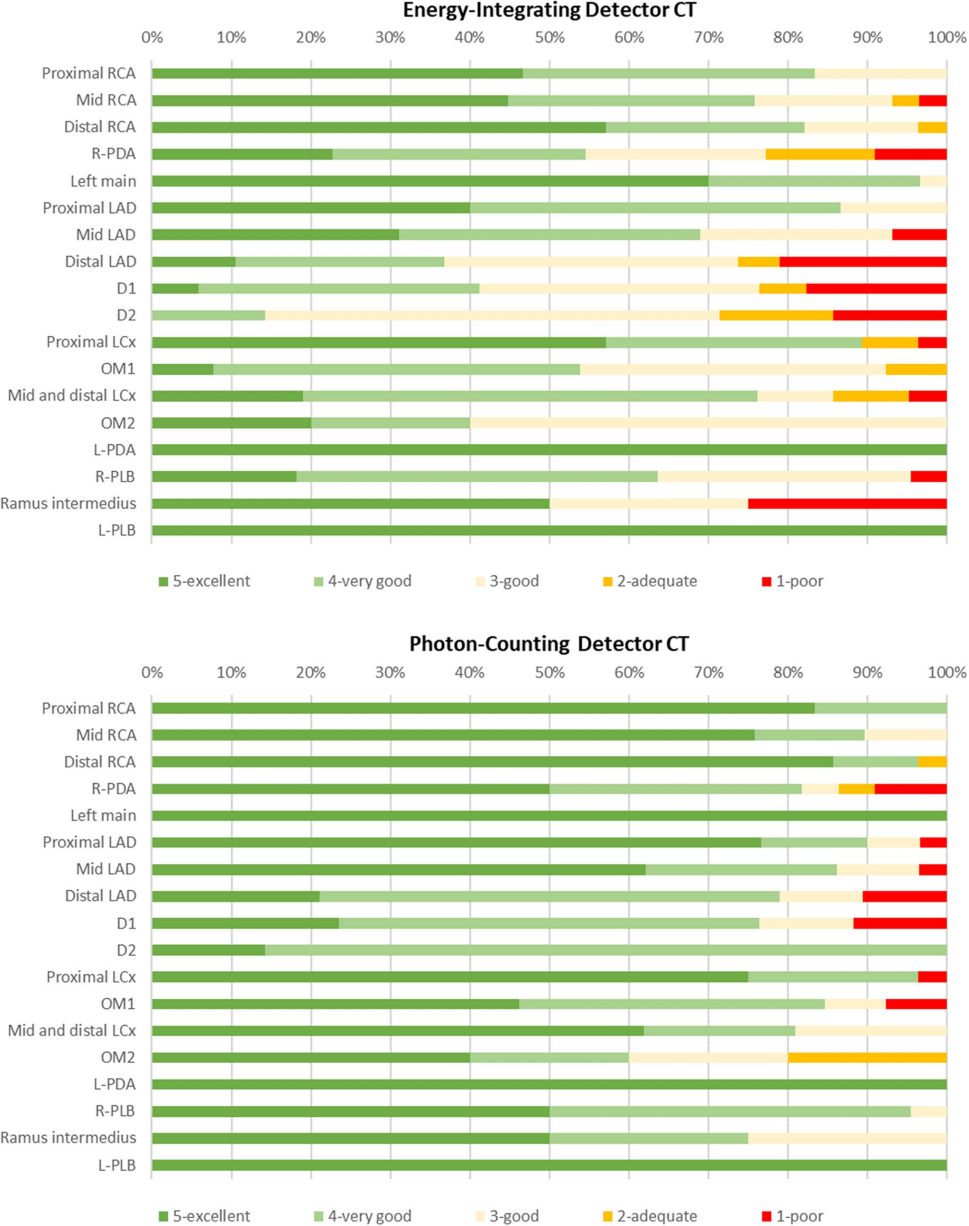
Image quality assessment of coronary segments in 30 patients: comparison between energy-integrating detector CT and photon-counting detector CT. The x-axis represents the percentage of coronary segments, ranging from 0 to 100%.. The y-axis represents the 18 different coronary segments. Each segment is divided into coloured sections, with the size of each section corresponding to the percentage of that segment with a particular quality score. The colour of each section represents the corresponding quality score (graded using the 5-point Likert scale). Proximal RCA = Proximal Right Coronary Artery; Mid RCA = Mid Right Coronary Artery; Distal RCA = Distal Right Coronary Artery; R-PDA = Right Posterior Descending Artery; Left main = Left Main Coronary Artery; Proximal LAD = Proximal Left Anterior Descending Artery; Mid LAD = Mid Left Anterior Descending Artery; Distal LAD = Distal Left Anterior Descending Artery; D1 = First Diagonal Branch of the LAD; D2 = Second Diagonal Branch of the LAD; Proximal LCx = Proximal Left Circumflex Artery; OM1 = First Obtuse Marginal Branch of the LCx; Mid and distal LCx = Mid and Distal Left Circumflex Artery; OM2 = Second Obtuse Marginal Branch of the LCx; L-PDA = Left Posterior Descending Artery; R-PLB = Right Posterolateral Branch; Ramus intermedius = Ramus Intermedius; L-PLB = Left Posterolateral Branch

**Fig. 2:**
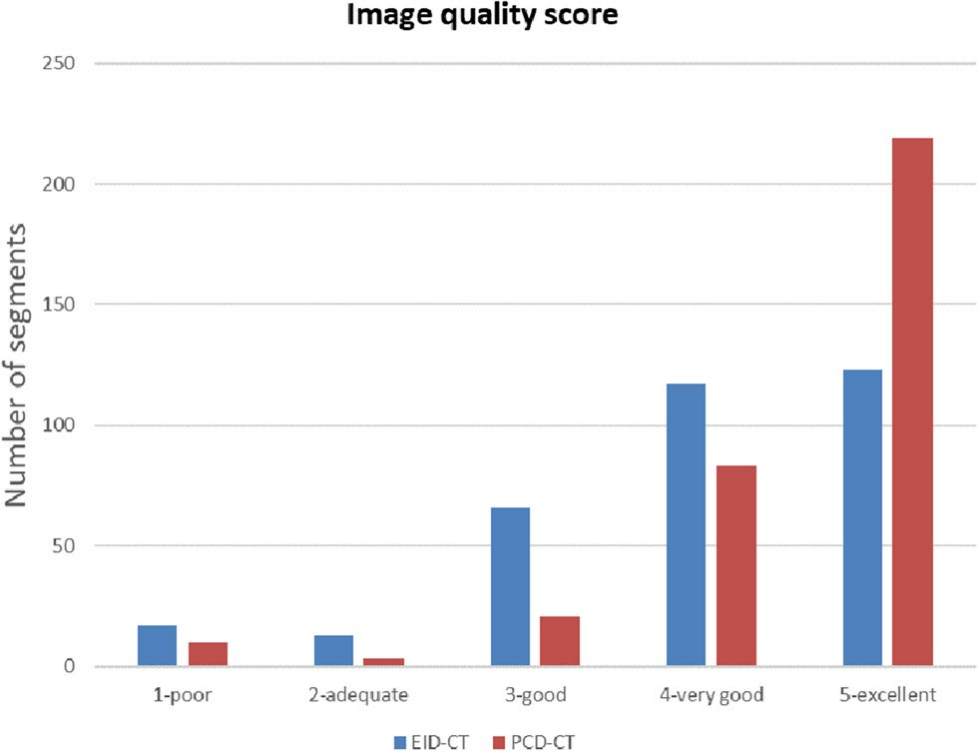
Comparison of image quality between energy-integrating detector CT and photon-counting detector CT

On the EID-CT scanner, 240 of 366 segments (71%) were rated as very good or excellent in quality compared to 302 of 336 segments (90%) on the PCD-CT scanner (*p* < 0.001). Image quality scores are presented in Table 2; Figs. 1 and 2.

**Table 2:** Comparison of image quality, stenosis degree, and plaque characteristics per segment between Energy-Integrating detector CT and Photon-Counting detector CT

	EID-CT (*N* = 336)	PCD-CT (*N* = 336)	*P* value
**Evaluable segments for image quality**			
1 – Poor IQ score	17 (5%)	10 (3%)	< 0.001
2 – Adequate IQ score	13 (4%)	3 (1%)	
3 – Good IQ score	66 (20%)	21 (6%)	
4 – Very good IQ score	117 (35%)	83 (25%)	
5 – Excellent IQ score	123 (37%)	219 (65%)	
**Evaluable segments for degree of stenosis**			
No luminal narrowing	243 (72%)	239 (71%)	0.941
1–24%	43 (13%)	43 (13%)	
25–49%	16 (5%)	26 (8%)	
50–69%	2 (1%)	6 (2%)	
70–89%	2 (1%)	4 (1%)	
≥ 90%	1 (< 1%)	2 (1%)	
Non-evaluable	29 (9%)	16 (5%)	
**Number of segments with plaque**	64 (19%)	81 (24%)	0.010
Calcified plaque	40 (12%)	33 (10%)	0.391
Non-calcified plaque	13 (4%)	10 (3%)	0.678
Mixed plaque	11 (3%)	38 (11%)	< 0.001

Data is presented as frequencies (percentage). Energy-Integrating Detector CT (EID-CT). Image quality (IQ). Photon-Counting Detector CT (PCD-CT)

Of the 336 evaluable segments per scan motion artifacts were present in 31 segments (9%) on EID-CT and 17 segments (5%) on PCD-CT (*p* = 0.040). Stack artifacts were observed in 29 segments (9%) on EID-CT and 38 segments (11%) on PCD-CT (*p* = 0.263).

**Fig. 3:**
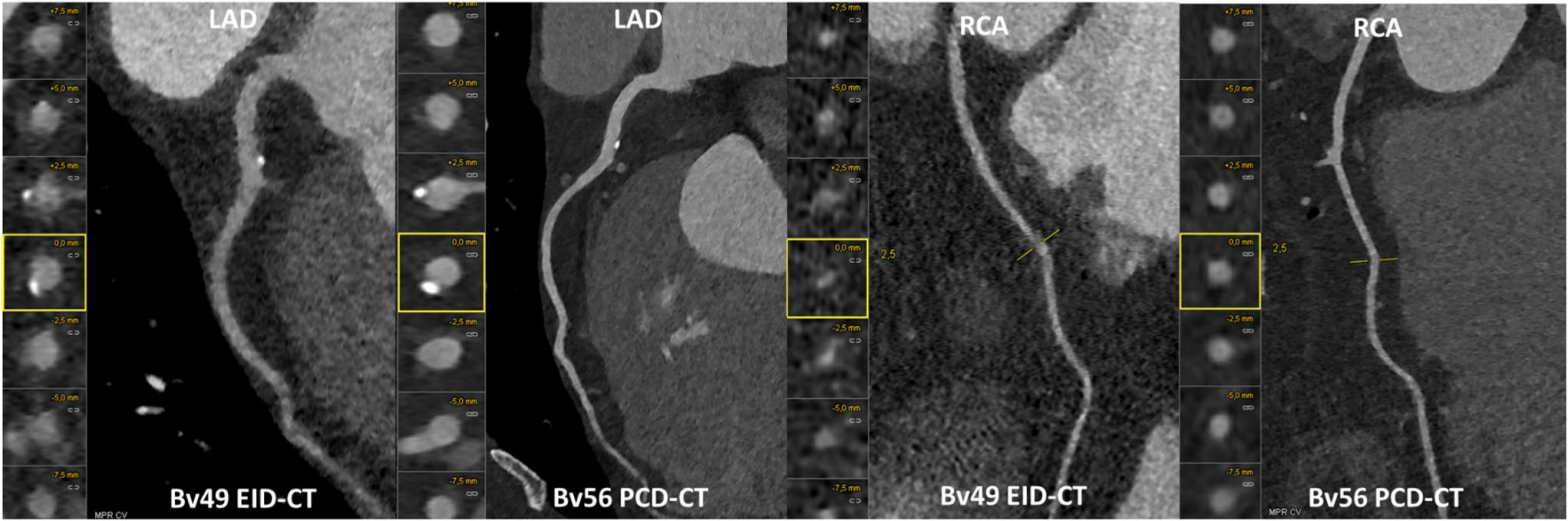
Multiplanar reconstructions of the coronary arteries of a 65-year-old post-heart transplantation patient using energy-integrating detector CT and photon-counting detector CT. On energy-integrating detector CT (EID-CT), the proximal segment of the left anterior descending artery displays a small calcification with an image quality score of 4-very good. On photon-counting detector CT (PCD-CT), the calcification is characterized as a mixed plaque (non-calcified plaque surrounding the calcification) with an image quality score of 5-excellent. For the right coronary artery, the entire artery was scored as 5-excellent with no plaque on PCD-CT. On EID-CT, the proximal right coronary artery segment was scored as 3-good with non-calcified plaque, while the mid and distal segments were rated as 2-adequate

**Fig. 4:**
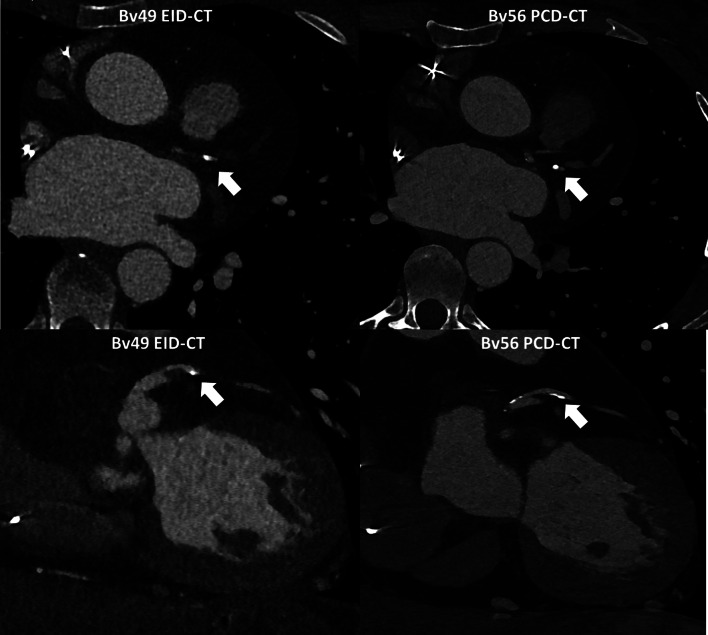
Assessment of coronary stenosis in a 31-year-old heart transplant patient using energy-integrating detector CT and photon-counting detector CT. 31-year-old heart transplant patient with a 50–69% stenosis observed in the obtuse marginal artery on the energy-integrating detector CT (0.6 mm slice thickness, Bv49 kernel). On photon-counting detector CT (0.2 mm slice thickness, Bv56 kernel), the stenosis was downgraded to 25–49%, indicating it was not significant. This example demonstrates that photon-counting detector CT provides enhanced diagnostic confidence compared to energy-integrating detector CT

On EID-CT, the degree of stenosis was non-evaluable for 29 of 336 segments (9%), and 64 (19%) demonstrated luminal narrowing. On PCD-CT, the degree of stenosis was non-evaluable for 16 of 372 segments (5%), and 81 segments (24%) showed luminal narrowing. There were significantly more segments with coronary plaques identified on the PCD-CT compared to the EID-CT (24% vs. 19%, *p* = 0.010) and this was mainly due to more mixed plaques (11% vs. 3%, *p* < 0.001) (Table 2). Figures 3 and 4 illustrate example cases of HTX patients scanned with both EID-CT and PCD-CT.

### Objective image quality assessment

The mean SNR was significantly higher on PCD-CT than EID-CT (22.1 ± 6.1 vs. 14.3 ± 3.4, *p* < 0.001). Similarly, the mean CNR was significantly higher on PCD-CT than on EID-CT (27.8 ± 6.8 vs. 16.9 ± 3.6, *p* < 0.001).

## Discussion

This study aimed to compare the image quality of EID-CT in comparison to PCD-CT for coronary artery visualization in HTx patients. Our results demonstrate that PCD-CT significantly enhances image quality, with 90% of coronary segments rated as very good or excellent on PCD-CT, compared to 71% on EID-CT. Furthermore, PCD-CT allowed for the evaluation of more coronary segments than EID-CT. PCD-CT also showed improvements in SNR and CNR. These findings suggest that PCD-CT provides superior coronary visualization, potentially aiding in detecting and monitoring CAV in HTx patients.

Our findings align with previous studies showing PCD-CT to provide superior image quality over EID-CT in coronary imaging. Pinos et al. reported higher CNR values and improved subjective image quality with PCD-CT compared to EID-CT [18]. Similarly, the study by Si-Mohammed et al. demonstrated that PCD-CT offers enhanced image quality and diagnostic confidence compared to EID-CT [19]. In our study, diagnostic confidence also appeared higher with PCD-CT, as fewer segments were rated as non-evaluable due to size or absent on PCD-CT compared to EID-CT.

In our study, the time between the EID-CT and PCD-CT scans was 17 months. Given the progressive nature of CAV, an increase in the percentage of segments with luminal narrowing over this period would be expected. However, we observed a modest increase, with 19% of segments showing luminal narrowing on EID-CT and 24% on PCD-CT. Studies that performed same-day comparisons of EID-CT and PCD-CT, such as Koons et al., reported that ultra-high-resolution PCD-CT reduced percent diameter stenosis by an average of 11% compared to EID-CT [12]. Similarly, McCollough et al. found that PCD-CT led to lower visual estimates of percent stenosis than EID-CT [13]. The progressive time interval in our study likely contributed to the observed results. Additionally, the median coronary artery calcium score was higher at the time of the PCD-CT scan compared to the EID-CT scan. Despite this, PCD-CT maintained superior image quality, highlighting its robustness in assessing coronary arteries even with an increased coronary artery calcium burden.

A contributing factor to the improved image quality observed in PCD-CT scans is the higher radiation dose, which can be attributed to the use of higher kV settings (most patients were scanned at 120 or 140 kV), compared to the generally lower kV settings on EID-CT. While the increased dose associated with PCD-CT remains clinically acceptable, it represents a trade-off between radiation exposure and image quality. In future studies, assessing the feasibility of lower kV settings in PCD-CT could help reduce radiation dose while maintaining image quality, especially for HTx patients who require regular follow-up imaging [20].

Furthermore, PCD-CT identified a higher proportion of mixed plaques and a lower proportion of calcified plaques compared to EID-CT, where calcified plaques represented the majority and mixed plaques accounted for a smaller fraction. This shift in plaque composition suggests that the reduced blooming artifacts in PCD-CT enhance the visualization of non-calcified components within mixed plaques, allowing for more accurate plaque characterization and quantification and improving patient risk stratification. Mergen et al. demonstrated that the ultra-high-resolution mode with PCD-CT showed a reduction in the calcified plaque volume compared to reconstructions using 0.6 mm slice thickness [21]. These findings highlight the potential of PCD-CT to provide a more detailed assessment of plaque composition, which is particularly relevant in managing CAV in HTx patients. However, further validation with invasive imaging is required to confirm the clinical significance of these findings.

This study has several limitations. Firstly, the time interval between the scans may have influenced the comparisons due to the progressive nature of CAV. Even though we tried to minimize this effect by restricting the maximum time between scans to two years, some degree of disease progression remains unavoidable, potentially making the PCD-CT appear more sensitive to plaque burden. Secondly, no invasive coronary angiography was used as the reference standard, limiting the ability to assess the diagnostic accuracy of stenosis or plaque characterization findings. Thirdly, the sample size of our study is relatively small. Lastly, in our study, there were discrepancies in scan protocols, particularly differences in tube voltage settings between EID-CT and PCD-CT, which may have contributed to the observed differences in radiation dose and image quality. Future studies employing standardized scanning protocols (including standardised kV settings) and larger patient cohorts are necessary to validate these findings and further assess the clinical potential of PCD-CT in the follow-up of HTx patients.

In conclusion, this study demonstrates that ultra-high-resolution PCD-CT significantly improves image quality compared to EID-CT in HTx patients. With superior SNR, CNR, and reduced non-evaluable segments, PCD-CT offers enhanced coronary artery visualization, though at the cost of an increased radiation dose. These findings highlight the potential of PCD-CT to improve the evaluation of CAV in HTx patients.

## Electronic supplementary material

Below is the link to the electronic supplementary material.

Supplementary Material 1

## Data Availability

No datasets were generated or analysed during the current study.
