# Supplementary material for: Comparison of image quality between photon-counting detector CT and energy-integrating detector coronary CT angiography in heart transplant patients
Source: Int J Cardiovasc Imaging. 2025 Jun 2;42(3):393–401. doi: 10.1007/s10554-025-03433-7 (PMC12987789; doi:10.1007/s10554-025-03433-7)
Supplement: Supplementary file 1 — Supplementary Material 1 [file 10554_2025_3433_MOESM1_ESM.docx]

**Supplementary data**

**Table 1S** Scan protocol
